# Botany in Molecular Era: A Modern Science with Ancient Roots

**DOI:** 10.3390/ijms17030360

**Published:** 2016-03-10

**Authors:** Marcello Iriti

**Affiliations:** Department of Agricultural and Food Sciences, Milan State University, via G. Celoria 2, 20133 Milan, Italy; marcello.iriti@unimi.it; Tel.: +39-025-0316766; Fax: +39-025-036781

Botany—the study of plant life—is an ancient science. Since Theophrastus (371–287 BC), considered the “father of botany” and author of the two seminal treatises *On the History of Plants* and *On the Causes of Plants*, this science progressed with humanity, greatly contributing to its development and advancement: food plants are the nourishment for human and livestock, fibre plants are used for textile and paper production, medicinal plants represent an unlimited source of pharmacologically active ingredients whereas forestry species provide building material and fuel. In addition, plants, particularly rainforests, possess a valuable environmental significance as atmospheric carbon dioxide sinks, thus contributing to offset global climate changes.

Coming back to the history of botany, in the late 16th century, Robert Hooke inspired cell theory by observing, with an early microscope, the structure of cork, a tegumental plant tissue. In 1753, Carl Linnaeus published *Species Plantarum*, where established the binomial nomenclature, which has been used in the naming of all living organisms ever since.

Nowadays, modern botany is no more only a descriptive science, moving to the investigation of molecular and mechanistic aspects, and consists of a number of sub-disciplines: plant physiology, plant biochemistry, plant pathology, plant ecology, pharmaceutical botany, paleobotany, to name but a few. Therefore, now botany aims at investigating complex biological systems by an omics approach and bioinformatics tools for analysing and processing hundreds to thousands data (genes, proteins, and metabolites).

*Molecular Botany*, the new Section of the *International Journal of Molecular Sciences* (*IJMS*) [1], places in this briefly described scenario, inheriting the long-lasting Journal’s experience in the general field of plant biology, as attested by the Topical Collection “Plant Molecular Biology” [2] and related Special Issues, where 285 articles were published since 2011. As an academic editor, I am certain that the excellent heritage will be collected and continued, in order to meet, in the next years, the expectancies of both *IJMS* readers and authors.
